# Robot assisted radical nephrectomy + hysterectomy and specimen retrieval per vaginum (NOSE)

**DOI:** 10.1590/S1677-5538.IBJU.2018.0351

**Published:** 2019-07-27

**Authors:** Jagdeesh N. Kulkarni, Nitesh Maurya, Sushrut Bhukte, Vrunda Karanjgaokar

**Affiliations:** 1 Department of Urology, Bombay Hospital and Medical Research Centre, Mumbai, India; 2 Department of Surgical Oncology, Asian Cancer Institute, Mumbai, India; 3 Department of Uro-Oncology, Asian Cancer Institute, Mumbai, India; 4 Department of Gynae-Oncology, Asian Cancer Institute, Mumbai, India

## Abstract

We demonstrate robot assisted radical nephrectomy with hysterectomy in the same sitting position followed by specimen retrieval per vagina (NOSE- Natural Orifice Specimen Extraction)

A 36 year old female P2L2, presented with long standing dysmenorrhoea. Abdominal sonography detected incidental large left renal mass with a large fundal fibroid.

CT scan revealed 8cmx8cm mass arising from mid and lower zone of the left kidney without vascular invasion and lymphadenopathy with a large fundal fibroid. Rest of the adnexae were normal.

She underwent robot assisted left radical nephrectomy first in lateral docking position. After bagging the nephrectomy specimen, robot was dedocked. Later, the patient was put in lithotomy position and with central docking, and hysterectomy was completed. Both the specimens were retrieved through the vagina without compromising the oncological principles.

Patient had a smooth postoperatory recovery and discharged on postoperative day 2. Histopathology revealed RCC Furhman grade 4 while hysterectomy specimen showed fibroadenoma with adenomyosis. No adjuvant therapy was instituted and at 3 months patient is doing well.

We conclude that two organ excision and extraction of specimen through vagina (NOSE) using two arms is possible in selected cases with excellent outcome in terms of early return to work with minimal morbidity. Also. limited use of instruments augments reduction in treatment cost.

## ARTICLE INFO

**Available at: http://www.intbrazjurol.com.br/video-section/20180351_Kulkarni_et_al**

**Int Braz J Urol. 2019; 45 (video #12): 642-642**

